# Acting on past lessons and learning new ones

**DOI:** 10.7554/eLife.91607

**Published:** 2023-09-06

**Authors:** Eduardo L Franco, Diane M Harper

**Affiliations:** 1 eLife Cambridge United Kingdom

**Keywords:** COVID-19, cancer screening, prevention, public health response, pandemic, cancer treatment, epidemiological modelling

## Abstract

eLife has published a special issue containing articles that examine how cancer prevention, control, care and survivorship were impacted by the COVID-19 pandemic.

We have seen much new science emerge since January 2020, when the World Health Organization (WHO) declared COVID-19 to be a public health emergency of international concern. Indeed, according to PubMed, researchers have published more than 420,000 articles on COVID-19, SARS-CoV-2 and related topics to date. Moreover, a number of efficacious and safe vaccines and anti-virals were developed in a relatively short period of time. Researchers from many different fields – including virology, epidemiology, molecular biology, vaccinology, and infectious disease modelling – were involved in these efforts.

For those who worked on the global HIV/AIDS epidemic, which began in 1981, the COVID-19 pandemic brought a sense of déjà vu. Back in the early 1980s, with the support of funding agencies, entire academic teams in a wide range of fields reoriented their research programs in an effort to control the effects of the HIV/AIDS epidemic via multiple areas, including epidemiology, HIV pathogenesis, vaccine development, diagnostics and therapy. Four decades later, HIV infection is no longer a death sentence, but there is still a need for research into new treatments and approaches for prevention and disease management, and to ensure that existing treatments are made available to vulnerable populations in low- and middle-income countries. The HIV/AIDS epidemic also resulted in a great deal of new science (almost 500,000 articles), albeit spread out over a longer time frame than the articles published on COVID-19 to date.

There are other parallels. Much like the HIV/AIDS epidemic required new science, new social practices, and new disease-management skills, so did the COVID pandemic. The COVID-19 lockdowns resulted in the suspension of cancer-prevention activities (such as tobacco control and vaccinations for hepatitis B and human papillomavirus) and prevented people in many countries from being screened for cancer. Treatments for patients with newly diagnosed cancers were also delayed, and existing patients – who were already at increased risk of death from the combined comorbidity of their cancer and COVID – had their oncology surgeries postponed. At the same time, like all other patients, cancer patients had to share their physicians with large numbers of COVID-19 patients: the pandemic also led to high levels of stress and burnout among medical staff, which has resulted in many leaving the profession. Understanding the impact of the pandemic on the entire trajectory of cancer – prevention, screening, diagnosis, therapy, survivorship, and end-of-life care – will help us plan interventions and prioritize care to mitigate or prevent the increases in cancer burden that may happen in the medium and long term.

There are also parallels in how the public and politicians responded to HIV/AIDS and COVID-19. Much like we learned to fight the pervasive bigotry that marginalized gay communities in the 1980s, we must learn how to fight the misinformation and disinformation that have hindered efforts to prevent the spread of COVID-19 in many countries. One stark difference between HIV/AIDS and COVID-19 is that we still do not have an effective vaccine against HIV.

The articles included in this special issue cover many different aspects of the impact of the COVID-19 pandemic on cancer across the globe, including both high- and low-income regions and countries. Many of the articles report the results of empirical research studies that captured the extent of the disruptions in care (such as disruptions in screening and vaccination activities, and delays in diagnoses and care). There are also articles about therapeutic interventions for the care of cancer patients affected by COVID-19, and articles about insightful modelling studies that provided projections of the impact of the pandemic on cancer prevention, control, and care pathways.

One of the lessons learned during the pandemic was that cancer control programmes could be made more resilient by taking advantage of recent technological advances: examples of this include the wider use of patient-centred screening for a number of cancers. The pandemic also confirmed the usefulness of videoconferencing for many different activities, including telemedicine, university teaching and virtual clinical appointments.

We thank all the reviewing editors, guest editors and reviewers who were involved in the peer review of the articles in this special issue (their names are on the home page for the special issue and/or in the individual articles), and we hope that it will serve as a valuable source of scientific evidence and information for those working in public health and elsewhere to develop plans to respond to future epidemics and pandemics. We also hope that this collection of articles will be a valuable historical account of what was often an improvised – yet innovative – response to a major public health threat.

Four decades after the start of the HIV/AIDS epidemic, HIV infection and AIDS are still with us. It is likely that SARS-CoV-2 and COVID-19 – and their successors – will also be with us for years to come, and that they will continue to inspire research that can be translated to provide new medicines, diagnostics and treatments. As the articles in this special issue confirm, it is essential that this research also includes work on managing cancer risk during any future public health emergency.

